# Intravenous Iron Carboxymaltose as a Potential Therapeutic in Anemia of Inflammation

**DOI:** 10.1371/journal.pone.0158599

**Published:** 2016-07-12

**Authors:** Niklas Lofruthe, Inka Gallitz, Lisa Traeger, Nicole Bäumer, Isabell Schulze, Tanja Kuhlmann, Carsten Müller-Tidow, Andrea U. Steinbicker

**Affiliations:** 1 Department of Anesthesiology, Intensive Care and Pain Medicine, University Hospital Muenster, University of Muenster, Muenster, Germany; 2 Department of Medicine A, Hematology, Oncology and Pneumology, University Hospital Muenster, Muenster, Germany; 3 Institute for Neuropathology, University Hospital Muenster, University of Muenster, Muenster, Germany; Lady Davis Institute for Medical Research/McGill University, CANADA

## Abstract

Intravenous iron supplementation is an effective therapy in iron deficiency anemia (IDA), but controversial in anemia of inflammation (AI). Unbound iron can be used by bacteria and viruses for their replication and enhance the inflammatory response. Nowadays available high molecular weight iron complexes for intravenous iron substitution, such as ferric carboxymaltose, might be useful in AI, as these pharmaceuticals deliver low doses of free iron over a prolonged period of time. We tested the effects of intravenous iron carboxymaltose in murine AI: Wild-type mice were exposed to the heat-killed *Brucella abortus* (BA) model and treated with or without high molecular weight intravenous iron. 4h after BA injection followed by 2h after intravenous iron treatment, inflammatory cytokines were upregulated by BA, but not enhanced by iron treatment. In long term experiments, mice were fed a regular or an iron deficient diet and then treated with intravenous iron or saline 14 days after BA injection. Iron treatment in mice with BA-induced AI was effective 24h after iron administration. In contrast, mice with IDA (on iron deficiency diet) prior to BA-IA required 7d to recover from AI. In these experiments, inflammatory markers were not further induced in iron-treated compared to vehicle-treated BA-injected mice. These results demonstrate that intravenous iron supplementation effectively treated the murine BA-induced AI without further enhancement of the inflammatory response. Studies in humans have to reveal treatment options for AI in patients.

## Introduction

Anemia is with more than 2 billion people affected worldwide one of the major public health burdens. The most common form of anemia is iron deficiency anemia (IDA). The second most common form is called anemia of inflammation (AI) or anemia of chronic disease. AI can develop in a previously healthy individual or in addition to an already existing IDA in response to increased cytokine levels and inflammation [1, 2]. The inflammatory cytokine interleukin-6 (IL-6) increases expression of the iron regulatory hormone hepcidin [3, 4, 5, 6]. Hepcidin, in turn, binds to the sole known iron exporter ferroportin-1 and thereby induces its internalization and degradation [7]. Ferroportin-1 is responsible for intestinal absorption of dietary iron and the release of iron from intracellular stores in enterocytes, macrophages, and hepatocytes [8, 9]. As a consequence, induction of hepcidin expression traps iron inside the iron storage cells and prevents intestinal iron absorption. Subsequently, serum iron levels decrease and AI occurs.

In intact iron homeostasis, high serum iron levels induce hepcidin [10, 11, 12]. Besides hepcidin, IL-6 also induces target genes such as the gene encoding superoxide dismutase 2 (SOD2) and hemeoxygenase 1 (HO-1) via phosphorylation of the transcription factor STAT3 [13]. Other cytokines also interfere with hepcidin regulation: Inhibition of the tumor necrosis factor α (TNF-α) in patients with rheumatoid arthritis and AI led to a decrease of hepcidin levels [14]. Furthermore, the monocyte chemoattractant protein 1 (MCP-1) release from macrophages correlates with high hepcidin levels [15].

A decrease in serum iron concentrations and impairment of erythropoiesis are known as protective mechanisms of the body during inflammation. Iron can be used by virus, bacteria or parasites for their replication and/or amplification and thereby might enhance the infectious disease [16, 17].

In patients with AI, treatment of the underlying disease is priority. The use of oral iron in inflammatory states is problematic, as oral iron cannot be absorbed from the gut or released from iron stores. Second, oral iron substitution might promote infections by delivering unbound iron or production of oxidative stress. A study in an endemic region for malaria in Zanzibar was interrupted, as routine oral iron and folate acid substitution caused an increase in overall mortality [18]. If indicated, severe AI is treated with red blood cell substitutes (RBCs), which may increase infections due to the release of unbound iron. A liberal transfusion practice led to an increase in severe hospital infections [19]. Intravenous iron supplementation in patients with AI is under investigation in clinical trials. If labile iron may increase the inflammatory response, the question arises, how pharmaceutically available high molecular weight complexes influence infections and inflammatory conditions.

The goal of the presented study was to investigate the efficiency of intravenous ferric carboxymaltose, a so called “type I” iron complex, to treat AI and the change of the inflammatory reaction in a murine model of AI. According to Geisser et al. type I complexes are described as robust with a long elimination half-time of 7–12h, strong in their kinetic and thermodynamic variability and should release labile iron in low amounts only [20]. Hypothetically low amounts of iron ions should be bound by transferrin directly and therefore be unavailable for infections and not be harmful in inflammatory settings.

In the current study, the effects of intravenous ferric carboxymaltose treatment were investigated in the established *Brucella abortus* (BA) AI mouse model which presents features similar to human AI, provoking a transient inflammation and AI [21, 22, 23]. In a short term experiment (4h BA followed by 2h intravenous iron), the effect on the acute inflammatory response under IDA conditions was analyzed. In a second set of experiments, mice were fed either a regular—or an iron deficient diet to model IDA—and injected with intravenous iron 14d after the intraperitoneal BA injection. Hematological and iron parameters as well as inflammatory markers were measured 24h and 7d after intravenous iron treatment.

We report that intravenous ferric carboxymaltose raised serum iron levels effectively and did not enhance the BA-mediated inflammatory response or caused an inflammatory response by itself. The diet influenced the duration until AI was treated: Intravenous iron treatment cured the AI within 24h in mice fed a regular diet, and within 7d in mice on an iron deficient diet.

To summarize, intravenous ferric carboxymaltose was able to effectively treat BA-induced AI without an augmentation of the inflammatory response provoked by BA itself.

## Materials and Methods

### Animal research ethics statement

This study was carried out in strict accordance with the recommendations and approval of the institutional ethics committee for “Animal Care of North Rhine-Westphalia, the *Landesamt fuer Natur*, *Umwelt und Verbraucherschutz (LANUV)*, North Rhine-Westphalia, Germany”, permit number Az.84-02.04.2013.A281. Blood withdrawal was performed under ketamine/xylazine anesthesia, and all efforts were made to minimize suffering. Under deep anesthesia, cervical dislocation was performed prior to organ collection.

### Heat-killed *Brucella abortus* mouse model

*Brucella abortus* (Strain99, *Brucella abortus* MRT AG PA0048) from c-c-pro GmbH (Oberdorla, Germany) was prepared as described by Sasu *et al*. [21].

Female WT C57BL/6 mice fed an iron-deficient diet (Altromin C1038, 5ppm iron) for 4 weeks were injected once with BA or PBS intraperitoneal. Intravenous ferric carboxymaltose (Ferinject^®^, 0.015mg/g body weight equivalent to maximum ferric carboxymaltose given in humans) [24] or PBS was given 4h after BA injection, blood and organs were harvested 2h later. In a second approach, mice were either fed an iron deficient diet or a regular diet (Altromin, 198ppm iron, Lage, Gemany) for 4 weeks prior to BA or PBS administration—and throughout the experiment. 14d after the BA administration, mice were treated intravenously with iron carboxymaltose (0.015mg/g body weight) or PBS. Blood samples, liver, and bone marrow were collected at 24h or 7d after intravenous iron or PBS administration. A chart of the experimental setup is provided in the supplement (S1 Fig). There was no difference in bodyweight within the groups of mice (S4 Fig)

### Hematologic and Iron Parameters

Blood samples were collected by retro-orbital puncture and serum iron parameters were determined as previously described [5]. Complete blood counts were obtained using the scil Vet abc Plus^™^ (Viernheim, Germany). Non-heme tissue iron levels were determined as previously described [25].

Reticulocytes were stained by adding 5μl blood to 1ml thiazole-orange reagent (Retic-COUNT^™^, BD Bioscience, San Jose, CA) for 1h at room temperature and measured by flow cytometry (FACSCalibur^™^, Becton Dickinson, Heidelberg, Germany). Unstained controls were used to exclude background fluorescence. Results are expressed as reticulocyte production index: RPI = Retic%xHb/14.46, with 14.46g/dL as the mean baseline hemoglobin (Hb) level of healthy WT mice.

### Hepatic mRNA levels

Total mRNA was isolated from liver tissue using Trizol^®^ (Sigma, Hamburg, Germany).

Semi-quantitative RT-PCR was performed on an Applied Biosystems 7500 Fast Real-Time-PCR system with LuminoCt^®^ SYBR^®^ Green qPCR ReadyMix^™^ (Sigma, Hamburg, Germany) using conventional primers (S1 Table). The relative C_T_ method was used to normalize the levels of target transcripts to 18S rRNA.

### Inflammatory serum cytokines

Inflammatory serum cytokines were determined using flow cytometry (BD FACSAria^™^ II flow cytometer, Heidelberg, Germany) and the CBA Mouse Th1/Th2/Th17 Cytokine Kit (BD Biosciences, Heidelberg, Germany) according to the manufacturer’s instructions.

The limit of detection is according to the manufacturer`s instructions as follows: IL-2 0.1 pg/ml, IL-4 0.03 pg/ml, IL-6 1.4 pg/ml, IFN-γ 0.5 pg/ml, TNF 0.9 pg/ml, IL-17A 0.8 pg/ml and for IL-10 16.8 pg/ml. The upper limit is 5000 pg/ml for each cytokine.

### Measurement of Erythroid progenitor cells

Bone marrow (BM) was collected and processed as described previously [26]. BM cells were stained with APC-conjugated rat anti-mouse CD44 and PE-conjugated TER119 rat anti-mouse (BD Pharmingen, Heidelberg, Germany) in PBS/2%FBS for 30min at RT. Analysis was performed using the BD FACSDiva^™^ software on a FACSCalibur^™^ (Becton Dickinson, Heidelberg, Germany). Unstained cells were used as background control.

Ter119+ events were considered as erythroid precursors which were further discriminated by cells size (forward scatter) and CD44 expression. CD 44 expression declines as erythroid precursors reach later stages of differentiation [27].

### Protein Analysis

Liver tissue samples were prepared as described before [5]. Extracted proteins were separated by electrophoresis using 4% to 10% bis-Tris gels and transferred to nitrocellulose membranes (Amersham, Germany). Membranes were incubated with antibodies directed against STAT3 phosphorylated at tyrosine705 (Cell Signaling Technology), and α-Tubulin (Cat. No. T6074, Sigma). ECL-Plus (Bio-Rad, Munich, Germany) was added and chemiluminescence was detected with a ChemiDocXRS+ (Bio-Rad, Munich, Germany).

### Prussian Blue Staining

Iron staining of paraffin-embedded liver sections was performed as previously described [28].

### Statistical analysis

Values are expressed as mean±SD. Data were analyzed using the Students *t*-Test and two-way ANOVA with Bonferroni's multiple comparisons test. Statistical significance was considered for p values <0.05.

## Results

### Intravenous iron supplementation increased hepcidin and serum iron levels in inflammation

**Fig 1:**
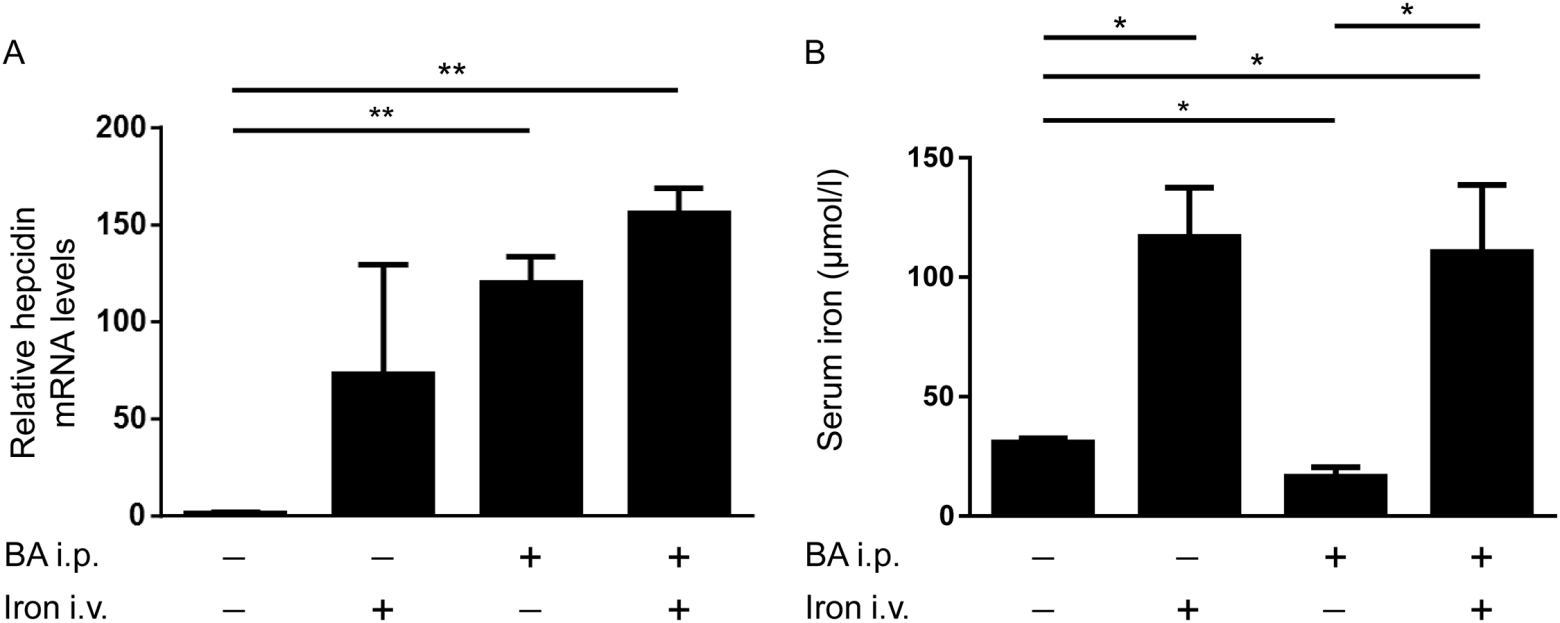
Induction of hepatic hepcidin mRNA levels and serum iron levels after intraperitoneal injection of BA and/or intravenous iron carboxymaltose administration. **(A)** Hepatic hepcidin mRNA levels were determined by quantitative RT-PCR in C57BL/6 mice 4h after intraperitoneal BA or PBS administration followed by intravenous iron or PBS treatment for an additional 2h (values represent mean±SD, n = 3, 2-way ANOVA, P = 0.002; **P = 0.004: PBS/PBS vs BA/PBS, and **P = 0.002: PBS/PBS vs BA/iron). **(B)** Serum iron levels were measured (values represent mean±SD, n = 3, 2-way ANOVA, P = 0.0008; *P = 0.02: PBS/PBS vs PBS/iron, *P = 0.01: PBS/PBS vs BA/PBS, *P = 0.04: PBS/PBS vs BA/iron, and *P = 0.03: BA/PBS vs BA/iron).

In order to estimate the effectiveness of intravenous iron to cure AI, WT mice were injected with or without BA for 4h followed by intravenous iron or vehicle application for an additional 2h. The administration of BA led to an induction of hepatic hepcidin mRNA levels after 6h independent of iron treatment (Fig 1A). Serum iron levels were reduced 6h after BA treatment (Fig 1B). Intravenous iron, in contrast, induced serum iron levels to a comparable extent, independently if mice were stimulated with or without BA (Fig 1B). These results indicate that intravenous iron was able to restore serum iron levels independent of BA-mediated hepcidin mRNA induction and BA-induced hypoferremia.

#### Intravenous iron did not enhance the BA-mediated increase in serum cytokines

**Fig 2:**
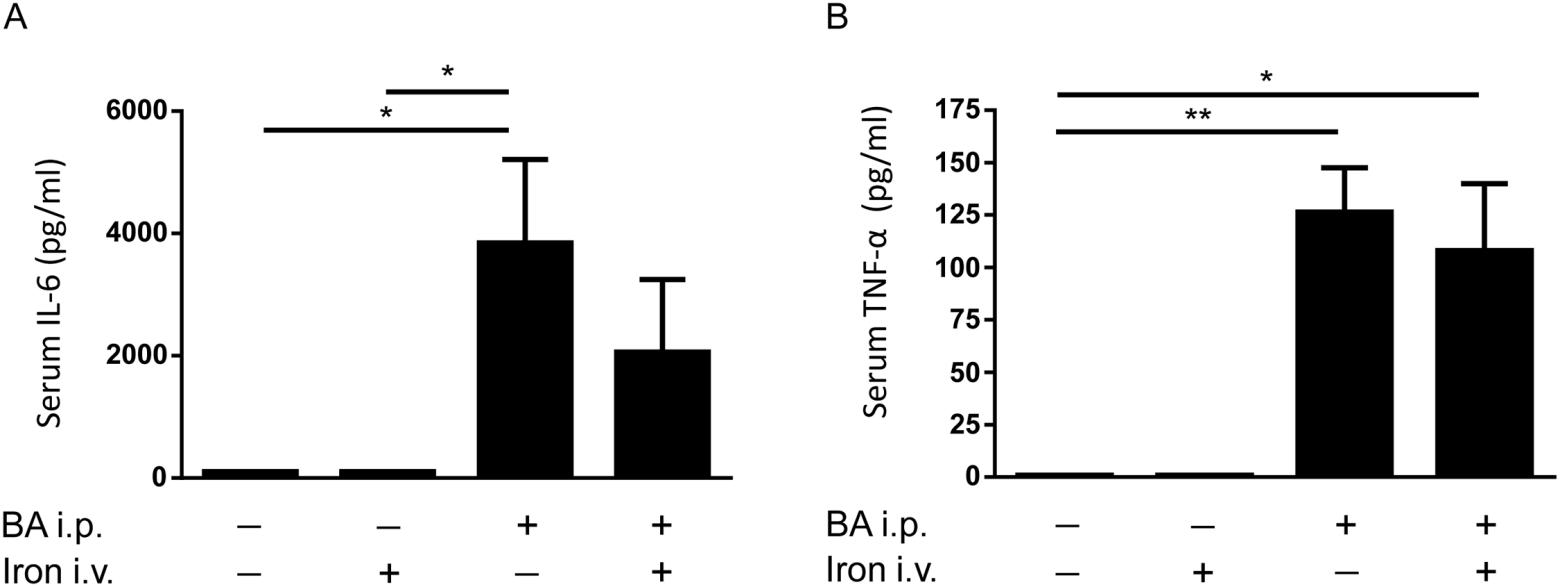
Serum cytokine levels measured after BA and intravenous iron administration. (**A**) Serum IL-6 protein levels were determined in C57BL/6 mice 4h after intraperitoneal BA or PBS administration followed by intravenous iron or PBS injection for an additional 2h (n = 3, 2-way ANOVA P = 0.003; *P = 0.04: PBS/PBS vs BA/PBS; *P = 0.04: PBS/iron vs BA/PBS). **(B)** Serum TNF-α levels (n = 3, 2-way ANOVA P = 0.03; **P = 0.009: PBS/PBS vs BA/PBS; *P = 0.03: PBS/PBS vs BA/iron; **P = 0.009: PBS/iron vs BA/PBS; *P = 0.03: PBS/iron vs BA/iron).

In order to determine the influence of BA-induced inflammation followed by intravenous iron carboxymaltose injection on the acute inflammatory response, serum cytokine protein levels were measured by flow cytometry using antibody coated beads. Upon an inflammatory stimulus, cells secrete pro-inflammatory cytokines such as IL-6. BA led to an increase in serum IL-6 protein levels after 6h as expected, whereas iron administration did not affect IL-6 levels (Fig 2A). TNF-α serum levels, as potential inducer of IL-6, were not elevated by intravenous iron treatment (Fig 2B). In contrast, BA injection, or BA plus intravenous iron injection, resulted in an increase of TNF-α serum protein levels in both groups (Fig 2B). These data indicate that intravenous iron did not enhance serum cytokine production induced by BA. The cytokines IL-2, IL-4, IL-10, IL-17A, and INF-γ were below the limit of detection (according to the manufacturer`s instructions between 0.03 pg/ml and 16.8 pg/ml).

#### Intravenous iron did not enhance the BA-mediated increase in hepatic markers of inflammation

**Fig 3:**
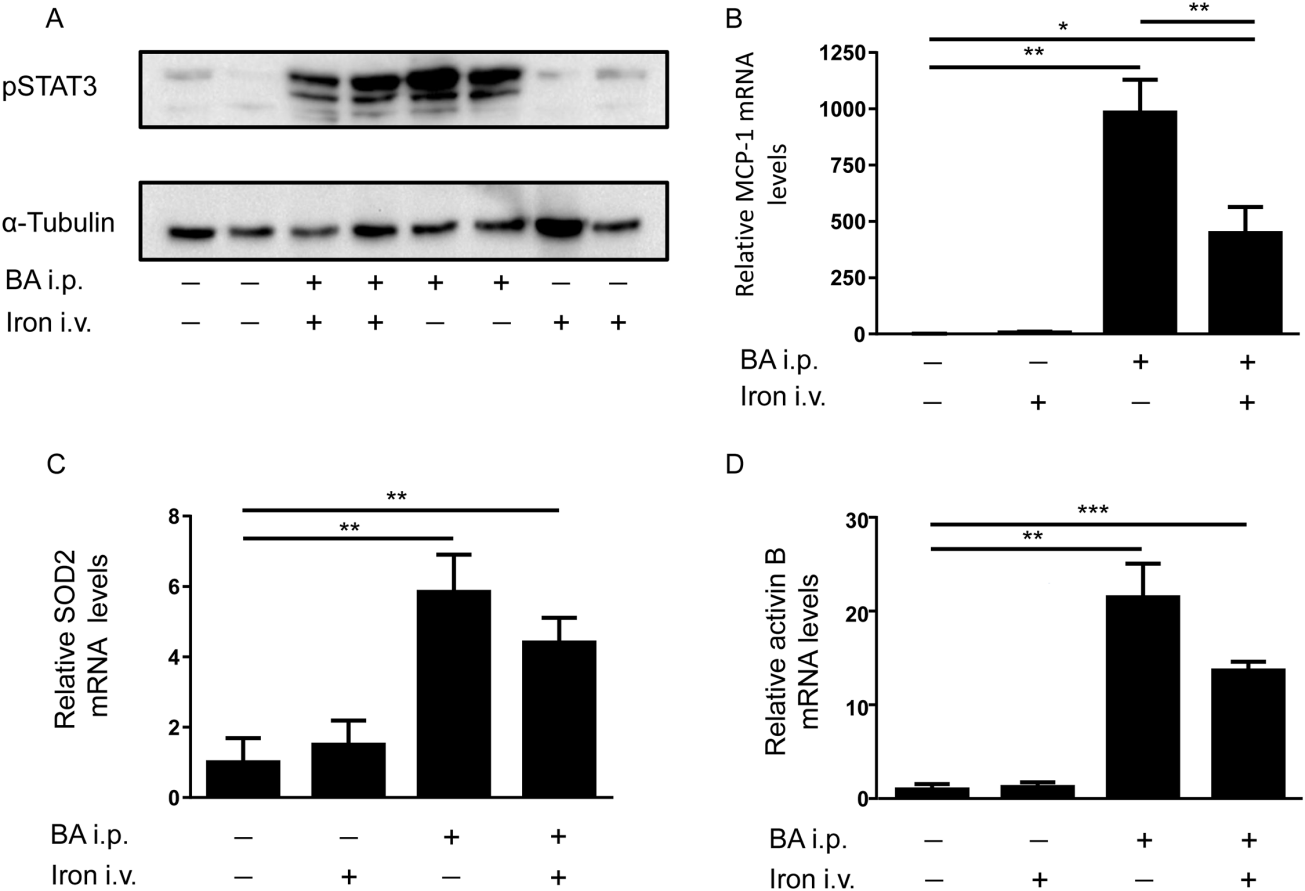
Hepatic mRNA levels of pSTAT3, MCP-1, SOD2 and activin B in the liver of WT mice after BA and intravenous iron injection. **(A)** Phosphorylated STAT3 levels compared to α-tubulin in the liver of C57BL/6 mice 4h after intraperitoneal BA or PBS administration followed by intravenous iron or PBS treatment for an additional 2h. **(B)** Hepatic MCP-1 mRNA levels were determined in WT mice after intraperitoneal BA or PBS administration followed by intravenous iron or PBS injection for an additional 2h (n = 3, 2-way ANOVA P<0,0001; **P = 0.007: PBS/PBS vs BA/PBS; *P = 0.02: PBS/PBS vs BA/iron; **P = 0.007: PBS/iron vs BA/PBS); (*P = 0.02: PBS/iron vs BA/iron; **P = 0.008: BA/PBS vs BA/iron; not shown in the graph). **(C)** Hepatic SOD2 mRNA levels in WT mice 4h after intraperitoneal BA or PBS administration followed by intravenous iron or PBS treatment for an additional 2h (n = 3, 2-way ANOVA P = 0.0002; **P = 0.004: PBS/PBS vs BA/PBS; **P = 0.004: PBS/PBS vs BA/iron); (**P = 0.006: PBS/iron vs BA/PBS; **P = 0.007: PBS/iron vs BA/iron, not shown in graph). **(D)** Hepatic activin B mRNA levels in WT mice 4h after intraperitoneal BA or PBS administration followed by intravenous iron or PBS injection for an additional 2h (n = 3, 2-way ANOVA P<0,0001; **P = 0.009: PBS/PBS vs BA/PBS; ***P = 0.0002: PBS/PBS vs BA/iron); (**P = 0.009: PBS/iron vs BA/PBS; ***P = 0.0003: PBS/iron vs BA/iron, not shown in graph).

To further analyze the effects of iron and BA treatment, the IL-6 signaling pathway in the liver was examined at protein and mRNA levels. Phosphorylation and thereby activation of the transcription factor STAT3 indicates IL-6 induction. Furthermore MCP-1 and SOD2 mRNA levels as markers of inflammation and oxidative stress were measured in the liver via qPCR analysis. The pro-inflammatory protein activin B, which is induced by lipopolysaccharides (LPS), is known to increase hepcidin mRNA levels via the BMP signaling pathway in mice. Iron treatment alone did not lead to STAT3 phosphorylation, MCP-1, SOD2 or activin B mRNA level changes compared to controls (Fig 3A–3D). Injection of BA, in contrast, led to STAT3 phosphorylation as well as an induction of MCP-1, SOD2 and activin B mRNA levels, which was not enhanced by subsequent iron treatment (Fig 3A–3D). Interestingly, iron administration significantly reduced the BA mediated MCP-1 mRNA induction (Fig 3B). To summarize, intravenous iron did not aggravate the BA-mediated inflammatory response.

### Intravenous iron administration accelerated recovery from BA-induced AI

**Fig 4:**
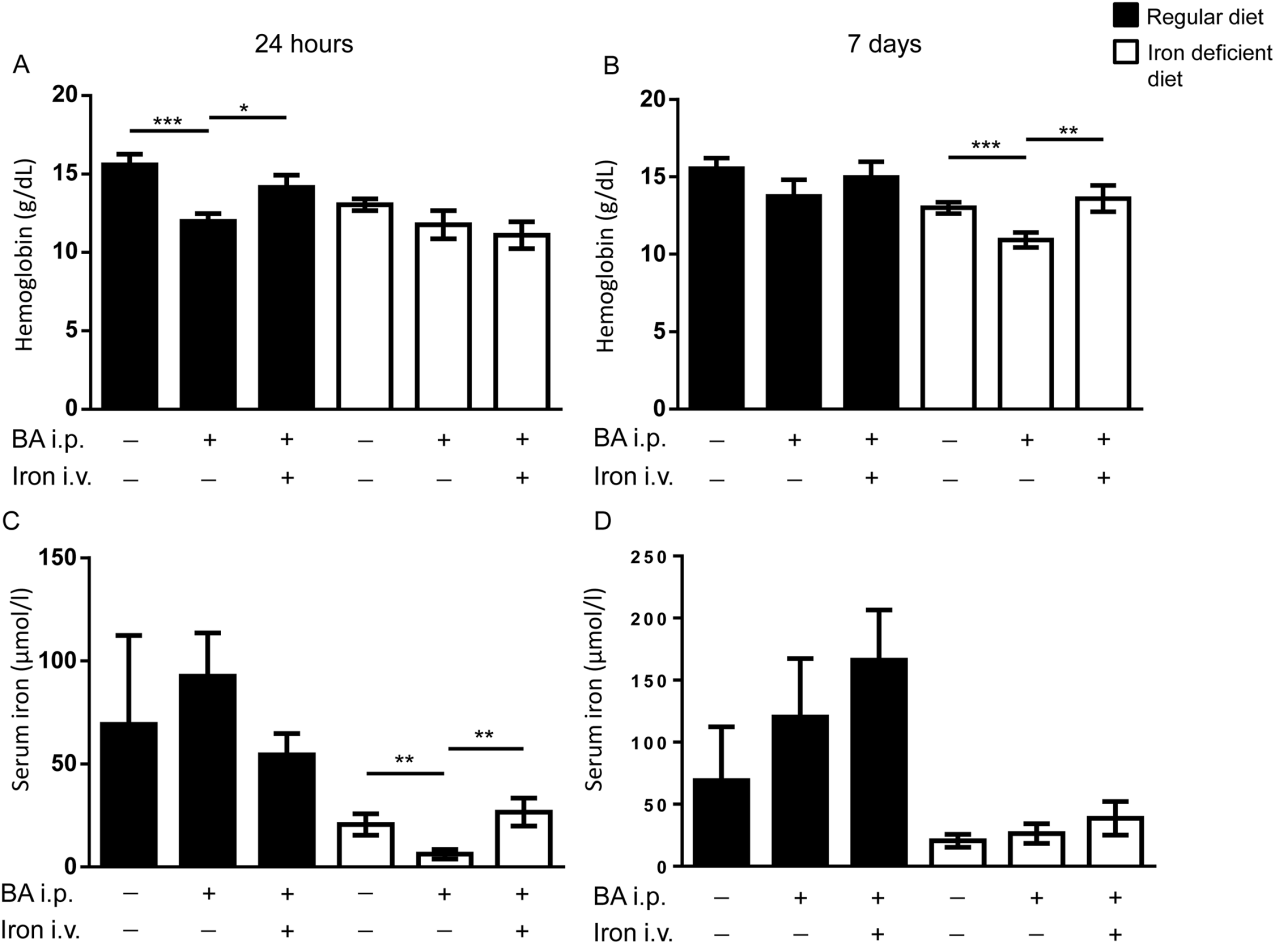
Intravenous iron treated BA-induced anemia in mice. WT mice were challenged with intraperitoneal BA injection and 14d later treated with intravenous iron. **(A)** Hemoglobin levels 24h after the iron treatment in mice fed a regular (black bars) or iron deficient (white bars) diet (regular diet: n = 3, 2-way ANOVA P < 0,0001; ***P = 0.0006: PBS/PBS vs BA/PBS; *P = 0.02: BA/PBS vs BA/iron). **(B)** Hemoglobin levels 7d after the intravenous iron treatment as in (A) (iron deficient diet: n = 4, 2-way ANOVA P = 0.002; ***P = 0.0007: PBS/PBS vs BA/PBS; **P = 0.003: BA/PBS vs BA/iron). **(C)** Serum iron levels 24h after intravenous iron treatment in mice on a regular or iron deficient diet (iron deficient diet: n = 4, 2-way ANOVA P = 0.007; **P = 0.006: PBS/PBS vs BA/PBS; **P = 0.006: BA/PBS vs BA/iron). **(D)** Serum iron levels 7d after intravenous iron treatment as in (C).

In order to analyze the influence of dietary iron on the BA model of AI, mice were either fed a regular diet or an iron deficient diet 4 weeks prior to and throughout the experiments. At day 14 after the BA injection the AI was treated either with a single dose of iron carboxymaltose or vehicle intravenously. Blood and organs were collected 24h or 7d after the intravenous iron treatment (experimental chart see S1 Fig). As the murine erythrocyte lifespan lasts 30–40 days, an increase in hemoglobin levels was expected 24 hours after intravenous iron substitution. Mice fed a regular diet developed BA-induced anemia with hemoglobin levels of 11.9±0.5g/dL compared to 15.6±0.7g/dL in control mice 24h after vehicle injection (Fig 4A). When treated with intravenous iron, hemoglobin levels increased within 24h to 14.1±0.8g/dL (Fig 4A). When fed a regular diet, mice recovered spontaneously from BA-induced AI 7d after vehicle injection with hemoglobin values of 13.7±1.1g/dL (Fig 4B). Therefore, hemoglobin levels were similar between controls, after BA administration, and after BA administration followed by iron treatment after 7d (Fig 4B).

Control mice under iron deficient conditions presented with hemoglobin levels of 13±0.4g/dL. BA-challenged mice fed an iron deficient diet decreased their hemoglobin levels to 11.7±0.9g/dL after 24h. When treated with intravenous iron, hemoglobin levels remained at 11±0.9g/dL in BA-challenged mice after 24h (Fig 4A). AI persisted 7d after vehicle treatment in BA-injected mice compared to control mice (10.9±0.5g/dL compared to 13±0.4g/dl, Fig 4B). Mice treated with intravenous iron presented with full recovery 7d after iron treatment when fed an iron deficient diet (13.6±0.9g/dL compared to 10.9±0.5g/dL in BA-injected mice). The data indicates that the availability of iron from the diet has an impact on the severity of anemia caused by BA administration.

Mice under iron restricted conditions presented IDA and developed prolonged AI-IDA. Intravenous iron helped to recover from the anemia at a later time point: 7d after intravenous iron when fed an iron deficient diet (AI on top of an IDA) compared to 24h in mice fed a regular diet (AI on a normal iron status).

While mice fed a regular diet presented with comparable serum iron levels (Fig 4C and 4D), mice fed an iron deficient diet displayed decreased serum iron levels after BA administration. 24h after intravenous iron treatment values were comparable to control mice (Fig 4C). 7d after iron therapy all three groups of mice presented with normal serum iron levels (Fig 4D).

In summary, intravenous iron restored serum iron levels. When serum iron levels were low due to an iron deficient diet (IDA-AI) prior to the inflammatory trigger, serum iron levels remained lower compared to control mice on a regular diet with intact iron stores (AI on a normal iron status).

### Iron treatment caused elevation of hepatic hepcidin mRNA levels in mice on iron deficient diet

**Fig 5:**
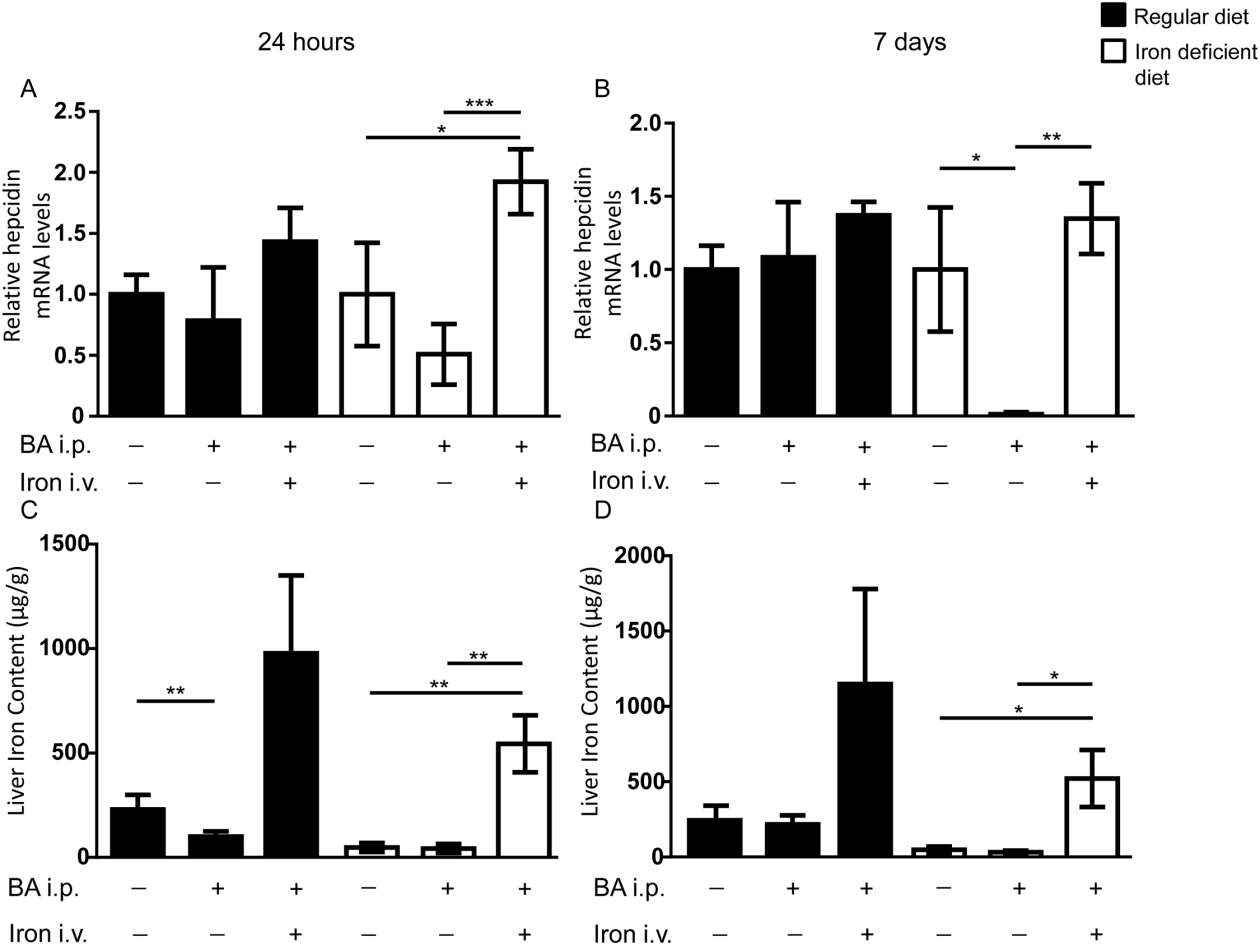
Hepcidin response to iron treatment depends on the diets. WT mice were challenged with or without intraperitoneal BA injection and 14d later treated with or without intravenous iron. **(A)** Hepatic hepcidin mRNA levels 24h after iron treatment in mice fed a regular iron diet (black bars) or an iron deficient diet (white bars) (iron deficient diet: n = 4, 2-way ANOVA P = 0.006; *P = 0.01: PBS/PBS vs BA/iron; ***P = 0.0002: BA/PBS vs BA/iron). **(B)** Hepcidin mRNA levels 7d after the iron treatment in mice as in (A) (iron deficient diet: n = 4, 2-way ANOVA P = 0.0007; *p = 0.02: PBS/PBS vs BA/PBS; **P = 0.001: BA/PBS vs BA/iron). **(C)** Liver iron content (LIC) was determined in C57BL/6 mice fed a regular or iron deficient diet. LIC 14d after BA and 24h after intravenous iron administration are shown (regular diet: n = 3–4, 2-way ANOVA P = 0.002; **P = 0.008: PBS/PBS vs BA/PBS, iron deficient diet: n = 4, 2-way ANOVA, P < 0.0001; **P = 0.005: PBS/PBS vs BA/iron; **P = 0.004: BA/PBS vs BA/iron). **(D)** LIC 14d after BA and 7d after iron treatment in mice fed a regular or an iron deficient diet (iron deficient diet: n = 4, 2-way ANOVA P = 0.0002; *P = 0.01: PBS/PBS vs BA/iron; *P = 0.01: BA/PBS vs BA/iron).

Mice fed a regular diet displayed no differences in hepatic hepcidin mRNA levels in control, BA*-*injected or BA-injected and iron treated mice at 24h or 7d (Fig 5A and 5B). In contrast, hepatic hepcidin mRNA levels of animals fed an iron deficient diet were increased 24h after intravenous iron treatment when compared to control or BA-injected mice (Fig 5A). BA-injected mice maintained on an iron deficient diet presented with reduced hepcidin mRNA levels after 7d compared to control mice (Fig 5B). In contrast, intravenous iron led to increased hepcidin mRNA levels after 7d (Fig 5B).

To conclude, normal body iron stores prevented an induction of hepcidin mRNA levels by intravenous iron or BA, while under iron deficient conditions an induction of hepcidin mRNA levels by iron after 24h could be observed.

#### Intravenous iron effectively increased tissue iron content in mice with AI

Mice fed a regular diet and treated with intravenous iron presented with a trend towards higher liver iron content compared to animals treated with PBS (Fig 5C and 5D). There was a reduction of liver iron content (LIC) in BA-injected control mice after 24h (Fig 5C). Mice fed an iron deficient diet presented with lower LIC than mice fed a regular diet.

Intravenous iron injection increased LICs comparably in BA-injected animals after 24h and 7d (Fig 5C and 5D, iron deficient diet 12.7x fold increase compared to mice fed a regular diet 10x fold increase). Histological staining revealed iron accumulation in the liver of mice treated with intravenous iron on regular diet or iron deficient diet. In mice injected with BA only, there was no iron accumulation (S2 Fig).

#### Induction of reticulocyte production by BA

**Fig 6:**
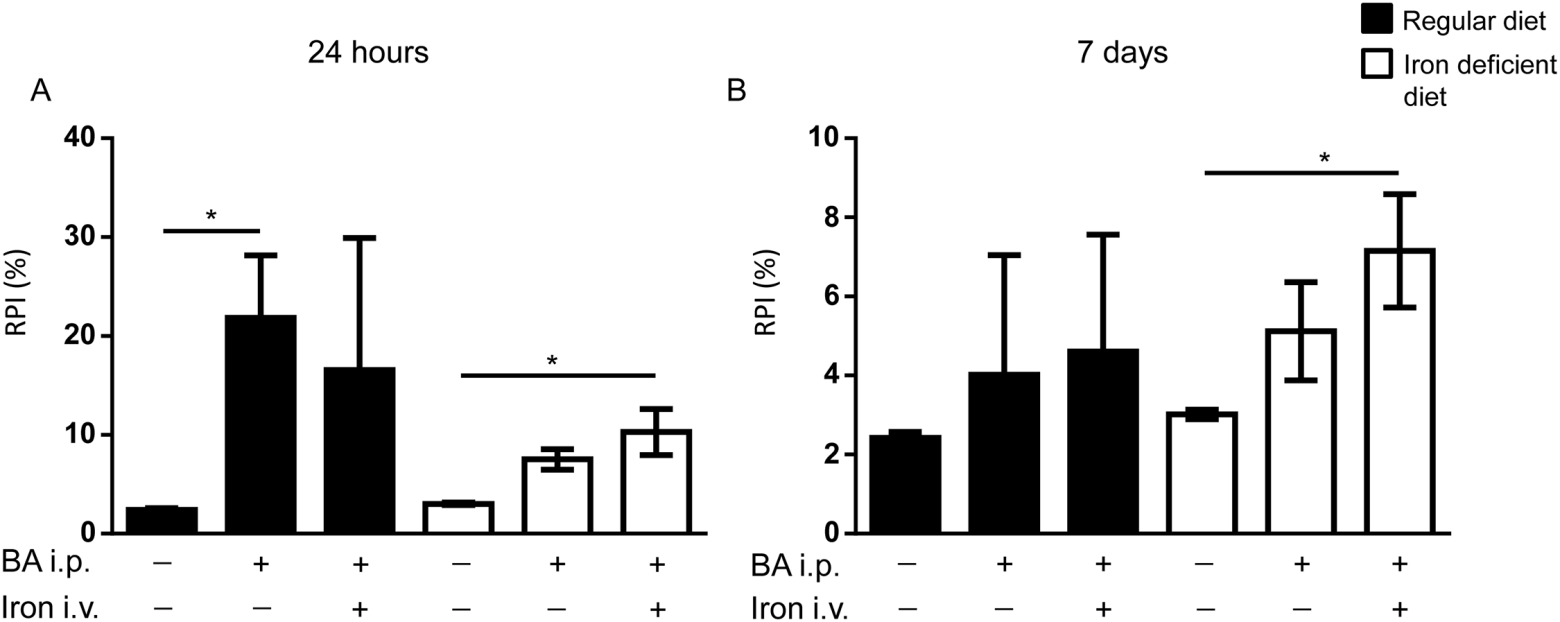
Reticulocyte production index (RPI) in BA-challenged mice fed a regular or an iron deficient diet and treated with intravenous iron or PBS. WT mice were fed a regular or iron deficient diet and injected with BA intraperitoneally followed 14d later by intravenous iron or PBS treatment. **(A)** Reticulocyte production Index (RPI = Retic%xHb/14.46, with 14.46g/dL as the mean baseline hemoglobin (Hb) level of healthy WT mice) 24h after iron or PBS treatment (2-way ANOVA P = 0.01, regular diet: n = 3, *P = 0.03: PBS/PBS vs BA/PBS; iron deficient diet: n = 3; *P = 0.02: PBS/PBS vs BA/iron). **(B)** Reticulocyte production 7d after iron or PBS treatment (iron deficient diet: n = 3–4, 2-way ANOVA P = 0.008; *P = 0.04: PBS/PBS vs BA/iron).

Reticulocyte production was assessed as reticulocyte production index (RPI). The RPI determines the response of the erythropoietic tissues to an anemic status. In mice fed a regular diet the RPI was elevated in response to BA-induced anemia (Fig 6A and 6B). Subsequent iron treatment did not induce the RPI further. However, a high variability was observed between individual animals within the groups (Fig 6A and 6B).

Under iron deficient conditions, BA-treatment plus vehicle as well as BA-treatment followed by intravenous iron application increased the RPI (Fig 6A and 6B). The data indicate that the response of erythropoietic tissues to BA-induced anemia is appropriate in mice fed a regular diet, whereas the response to BA-induced anemia in mice on an iron deficient diet was suppressed. Mice fed a regular diet were cured from anemia 24h after intravenous iron treatment. As a consequence the RPI was high at 24 hours and decreased at 7d. Intravenous iron improved the productivity of the erythropoietic tissue.

To further investigate the effect of intravenous iron treatment on bone marrow erythropoiesis, we performed FACS analysis of bone marrow cells to examine the amount of erythroid cells and their precursors. Total bone marrow cells were selected for Ter119^+^ expression to define erythroid cells.

To discriminate the erythroid precursors into subpopulations, cells were sorted by cell size (forward scatter) and CD44 expression. CD44 expression declines as erythroid precursors maturate. The total erythroid cell population (Ter119^+^ cells) in the bone marrow from mice fed a regular diet was neither affected by BA treatment nor by BA followed by iron treatment (S2A Fig). BA-injection led (after 24h) to a reduction of the terminal differentiated red cells (subpopulation V), which was abolished by intravenous iron treatment (S2B Fig). After 7d the terminal differentiated red cells remained lower than in controls. Mice fed an iron deficient diet presented an increase in erythroid cells 24h after intravenous iron treatment, an effect that was abolished after 7d (S3C Fig). The late stage subpopulation V was decreased at 24h and 7d (S3D Fig). The decrease was abolished by intravenous iron treatment after 24h, but not at 7d. All the differences appear to be small and similar in dot plots (data not shown).

Taken together, these findings indicate that late stage of erythropoiesis was impaired by BA treatment in all groups of mice injected with BA, which can be explained by an increase in cytokine levels. We conclude that albeit presence of inflammation, intravenous iron recovers the shift of the terminal differentiated red cell population.

## Discussion

The use of intravenous iron pharmaceuticals has to be carefully elucidated in AI in regard of two aspects: 1.) the ability to treat AI and 2.) enhancement of inflammation by intravenous iron. The current manuscript demonstrates that intravenous iron complex type I, ferric carboxymaltose, was effective to increase serum iron levels, tissue iron content and hemoglobin levels in the murine BA model of AI. The inflammatory reaction provoked by BA was neither acute nor chronically enhanced by intravenous iron. The data indicates that ferric carboxymaltose might be a therapeutic option in the murine BA-model of AI and in combined AI/IDA.

The BA model used in this study provokes a transient inflammatory reaction and is characterized by typical patterns of AI: anemia, inflammation, and iron restricted erythropoiesis. Models in rodents are useful to understand the pathophysiology of iron and infection, to observe dietary iron status, iron homeostasis and effects on erythropoiesis. However, the limitation of the study is that the BA model is a rodent model of ACD that cannot be transferred to humans directly. Additionally, heat-killed BA bacteria do not replicate. Studies using vital bacteria are required as a next step. Therefore the results do not indicate that intravenous iron can be used in human patients with inflammation and infection *per se*. Ferric carboxymaltose treated AI under two dietary conditions in the murine BA model: First, anemia of inflammation in mice on a regular diet (as AI may develop in healthy individuals characterized by normal iron stores), and second, in mice on an iron deficient diet 4 weeks prior to and throughout the experiment (as AI may develop on top of an IDA).

Due to the negative side effects of unbound iron—such as oxidative stress and enhanced inflammation—intravenous iron treatment has been controversially discussed in clinical trials [16]. The latter meta-analysis revealed that intravenous iron treatment increased the rate of infections in patients with an odds ratio of 1.33. A limitation of the study was that in the majority of studies, the rate of infections was analyzed retrospectively. Without doubt clinical trials in patients are needed.

In our experiments, intravenous iron treatment did not lead to an induction of SOD2 mRNA levels 2h after the iron injection (6h after BA injection). In another study, a sterile inflammation provoked by Zymosan (a glucan), or by E. coli lipopolysaccharide injection, did not induce hepatic SOD2 mRNA levels in control mice, but 4h after the iron injection [24]. The model is different from the BA model, as Zymosan activates the toll like receptor 2. In addition, the time point we chose in the current study was different, too. We aimed to detect BA-mediated changes in hepatic hepcidin mRNA levels, which reach a maximum 6h after the BA injection [23].

AI often occurs in patients in association with IDA. We therefore analyzed the effect of dietary iron restriction to model IDA prior to BA injection, on the recovery of AI and the effectiveness of intravenous iron to increase serum iron levels, iron stores and hemoglobin. While mice fed an iron deficient diet were still anemic 7d after vehicle treatment, mice on a regular diet already recovered spontaneously from AI at this time point. With iron deficient diet, the intravenous iron treatment led to a prolonged increase of hepcidin mRNA levels 24h and 7d after iron treatment. This effect occurred most likely due to the fact that high molecular weight iron complexes release iron slowly over time, thus presenting a long lasting iron source [20]. In contrast, hepcidin mRNA levels in mice fed a regular diet were not altered by intravenous iron. Heming et al. and Kim et al. used an iron deficient diet to enable the induction of hepcidin mRNA levels in mice by iron and/or an inflammatory stimulus [23, 24, 29]. Nemeth et al. concluded that the high iron content of standard rodent chow already maximally stimulates hepcidin mRNA expression and renders its ability to respond to an inflammatory stimulus [4]. These findings are in line with our results. The dietary iron supply had an impact on hepcidin expression and erythropoiesis. Mice fed an iron deficient diet and injected with BA displayed low hepcidin mRNA expression levels, presumably due to the suppressive effect of increased erythropoietic demand on the hepcidin expression.

The administration of heat-killed particles instead of vital/reproductive bacteria could lead to a diminished cytokine response. Nevertheless, it has been reported that inflammatory cytokines impair the bone marrow erythropoiesis [27, 30]. We demonstrate that a single administration of heat-killed BA led to an induction in cytokines and an impaired erythropoiesis. The enhanced reticulocyte production in BA-treated mice results from a mice specific splenic erythropoiesis, which was ineffective to prevent BA-induced anemia.

Clinically, anemia is with 30% prevalence a frequent co-morbidity, and if intravenous iron substitutes cannot be applied, blood transfusions are currently given to treat AI in hospitalized patients. With each red blood cell substitute labile iron is delivered to the patient. The dilemma is obvious and known: Red blood cell substitutes enhance the inflammatory condition of the patients.

## Conclusion

Taken together, intravenous iron was effective to increase serum iron levels, tissue iron content and hemoglobin levels in mice with AI with and without prior iron deficiency. Treatment of BA-induced AI with intravenous iron substitution did not increase the acute or the chronic inflammatory response. Therefore, treatment of AI with high molecular weight iron complexes such as ferric carboxymaltose, might be a therapeutic option for AI. However, further studies of intravenous iron treatment with high molecular weight complexes in other murine models (e.g. with viable and replicable pathogens) of AI, as well as in patients with acute or chronic inflammatory conditions, will have to be performed.

## Supporting Information

S1 Data Data summary short term murine BA model. (XLSX)

S2 Data Data summary longterm iron deficient diet murine BA model. (XLSX)

S3 Data Data summary longterm regular diet murine BA model. (XLSX)

S1 Fig Experimental design of BA experiments with different diets. WT mice were either fed a regular diet (198 ppm iron) or iron deficient diet (5ppm) 4 weeks prior to the administration of 5x10^8^ particles per mouse BA. After 14d mice were treated with a single dose (0.015 mg/g i.v.) iron carboxymaltose. 24h and 7d after the intravenous iron treatment blood and organs were collected.
(TIF)

S2 Fig Histological iron staining in the liver. Iron deposition in the liver of mice fed a regular or iron deficient diet after BA exposure and 7d after iron treatment. Formalin-fixed frozen sections of the liver stained with Prussian blue. **(A)** Mice fed a regular diet were treated with BA. **(B)** Mice fed a regular diet were treated with BA and subsequently with intravenous iron (7d). **(C)** Mice fed an iron deficient diet were treated with BA. **(D)** Mice fed an iron deficient diet treated with BA and subsequently treated with intravenous iron (7d).
(TIF)

S3 Fig Erythropoiesis in the bone marrow of mice fed a regular or iron deficient diet, and then exposed to BA followed by iron treatment. Erythroid maturation in the bone marrow was assessed by flow cytometry in C57BL/6 mice fed a regular iron diet or an iron deficient diet. Mice were then treated once with BA until anemic at 14d, followed by intravenous iron treatment for 24h or 7d. **(A)** The erythroid cell population (Ter119^+^) in the bone marrow (BM) of mice fed a regular iron diet is shown. **(B)** Erythroid precursors were analyzed according to size (forward scatter) and CD44 expression in mice fed a regular iron diet (n = 4, 2-way ANOVA P < 0.0001, *P˂0.05: V, PBS/PBS vs BA/PBS 24h; *P˂0.05: V, PBS/PBS vs BA/iron 7d). **(C)** The erythroid cell population (Ter119^+^) in the BM of mice fed an iron deficient diet are depicted (n = 4, 2-way ANOVA P = 0.0006; *P = 0.04: PBS/PBS vs BA/iron 24h; *P = 0.01: BA/iron 24h vs BA/iron 7d) **(D)** This figure shows the erythroid precursors in mice fed an iron deficient diet (n = 4, 2-way ANOVA P < 0,0001, ****P˂ 0.0001: V, PBS/PBS vs BA/PBS 24h; **P˂0.01: V, PBS/PBS vs BA/PBS 7d; *P˂0.05: V, PBS/PBS vs BA/iron 7d; **P˂0.01: V, BA/iron 24h vs BA/PBS 24h). [Subpopulation V = terminal differentiated red cells, IV = orthochromatic erythroblast, III = polychromatic erythroblast, II = basophilic erythroblast, I = proerythroblast].
(TIF)

S4 Fig Bodyweight. Bodyweight of WT mice were either **(A)** fed a regular diet (198 ppm iron) or **(B)** iron deficient diet (5ppm) 4 weeks prior to the administration of 5x10^8^ particles per mouse BA. After 14d mice were treated with a single dose (0.015 mg/g i.v.) iron carboxymaltose.
(TIFF)

S1 Table Quantitative real-time PCR primer. (TIF)
