# Supplementary figures and images for: Intravenous Iron Carboxymaltose as a Potential Therapeutic in Anemia of Inflammation
Source: PLoS One. 2016 Jul 12;11(7):e0158599. doi: 10.1371/journal.pone.0158599 (PMC4942094; doi:10.1371/journal.pone.0158599)

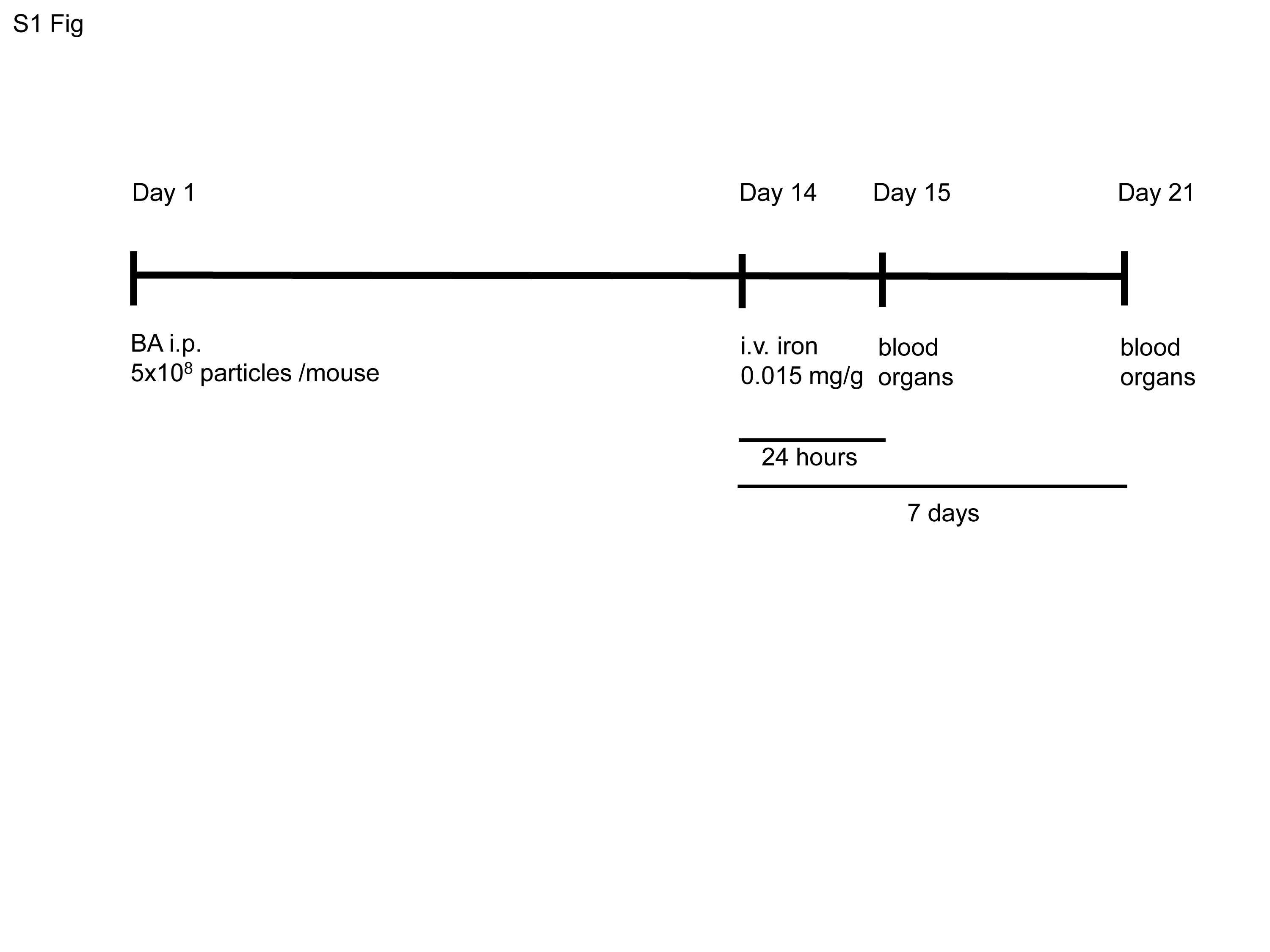

Supplement: S1 Fig — WT mice were either fed a regular diet (198 ppm iron) or iron deficient diet (5ppm) 4 weeks prior to the administration of 5x10^8^ particles per mouse BA. After 14d mice were treated with a single dose (0.015 mg/g i.v.) iron carboxymaltose. 24h and 7d after the intravenous iron treatment blood and organs were collected. (TIF) [file pone.0158599.s004.TIF]

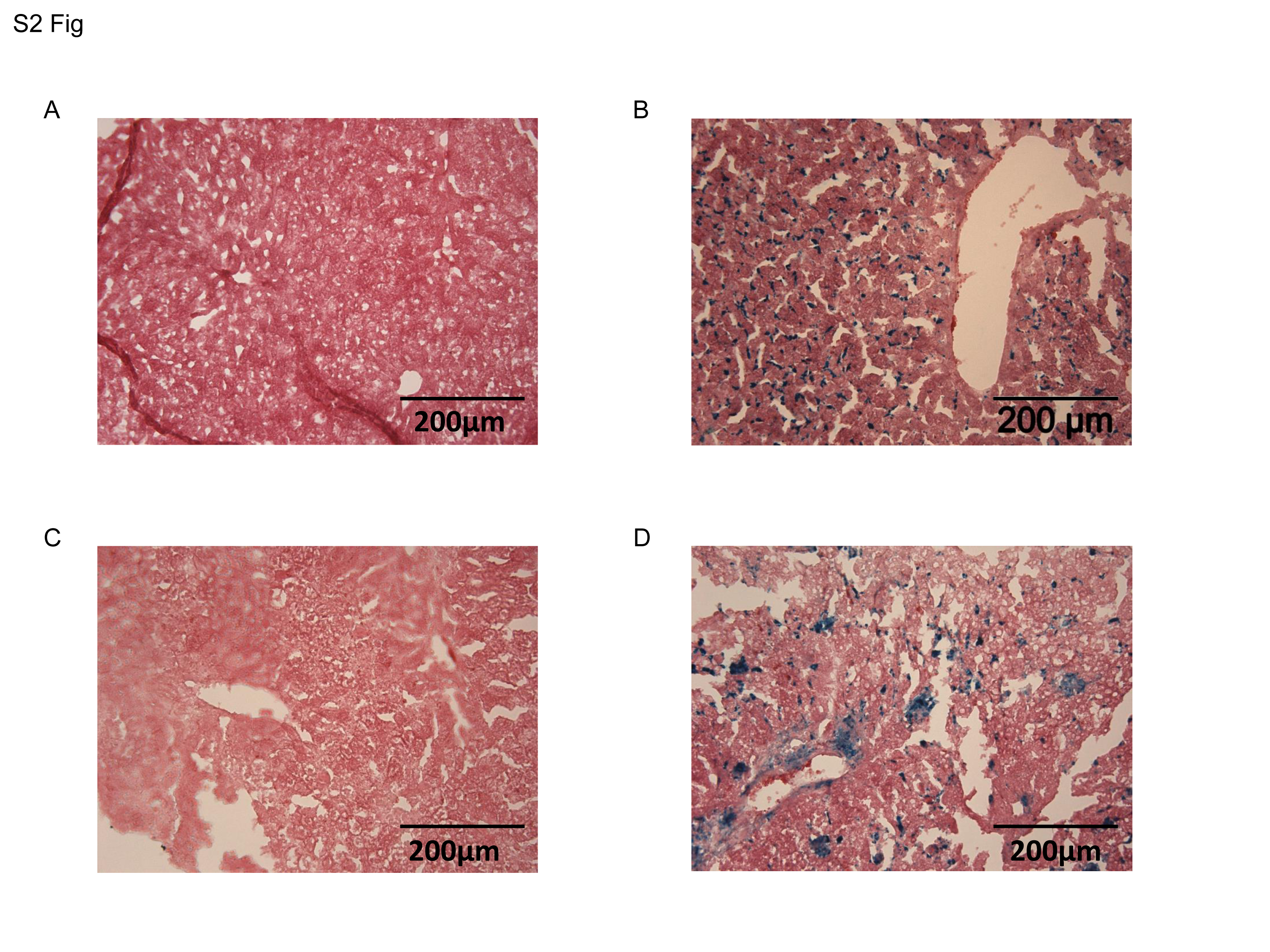

Supplement: S2 Fig — Iron deposition in the liver of mice fed a regular or iron deficient diet after BA exposure and 7d after iron treatment. Formalin-fixed frozen sections of the liver stained with Prussian blue. (A) Mice fed a regular diet were treated with BA. (B) Mice fed a regular diet were treated with BA and subsequently with intravenous iron (7d). (C) Mice fed an iron deficient diet were treated with BA. (D) Mice fed an iron deficient diet treated with BA and subsequently treated with intravenous iron (7d). (TIF) [file pone.0158599.s005.TIF]

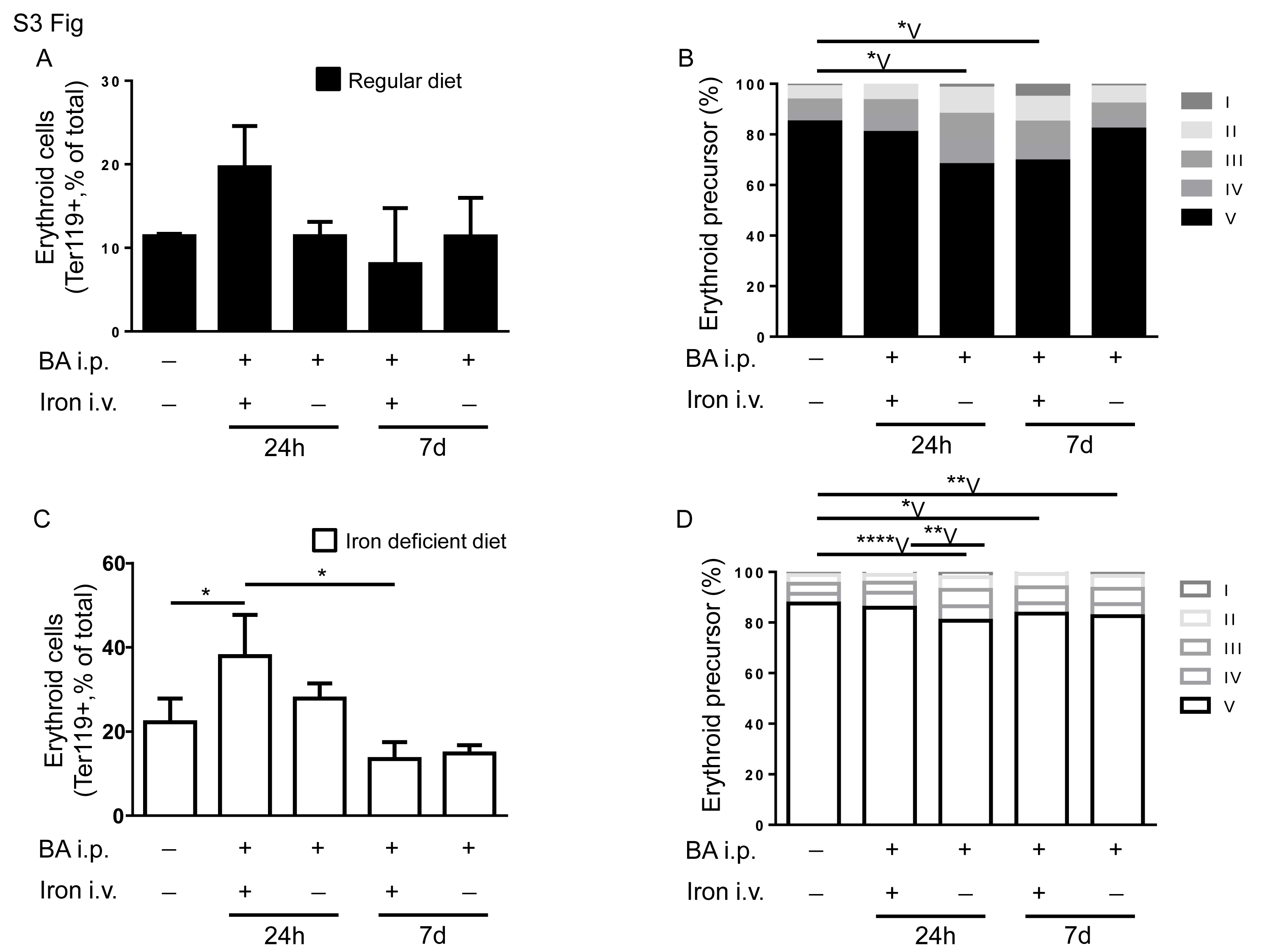

Supplement: S3 Fig — Erythroid maturation in the bone marrow was assessed by flow cytometry in C57BL/6 mice fed a regular iron diet or an iron deficient diet. Mice were then treated once with BA until anemic at 14d, followed by intravenous iron treatment for 24h or 7d. (A) The erythroid cell population (Ter119+) in the bone marrow (BM) of mice fed a regular iron diet is shown. (B) Erythroid precursors were analyzed according to size (forward scatter) and CD44 expression in mice fed a regular iron diet (n = 4, 2-way ANOVA P < 0.0001, *P˂0.05: V, PBS/PBS vs BA/PBS 24h; *P˂0.05: V, PBS/PBS vs BA/iron 7d). (C) The erythroid cell population (Ter119+) in the BM of mice fed an iron deficient diet are depicted (n = 4, 2-way ANOVA P = 0.0006; *P = 0.04: PBS/PBS vs BA/iron 24h; *P = 0.01: BA/iron 24h vs BA/iron 7d) (D) This figure shows the erythroid precursors in mice fed an iron deficient diet (n = 4, 2-way ANOVA P < 0,0001, ****P˂ 0.0001: V, PBS/PBS vs BA/PBS 24h; **P˂0.01: V, PBS/PBS vs BA/PBS 7d; *P˂0.05: V, PBS/PBS vs BA/iron 7d; **P˂0.01: V, BA/iron 24h vs BA/PBS 24h). [Subpopulation V = terminal differentiated red cells, IV = orthochromatic erythroblast, III = polychromatic erythroblast, II = basophilic erythroblast, I = proerythroblast]. (TIF) [file pone.0158599.s006.TIF]

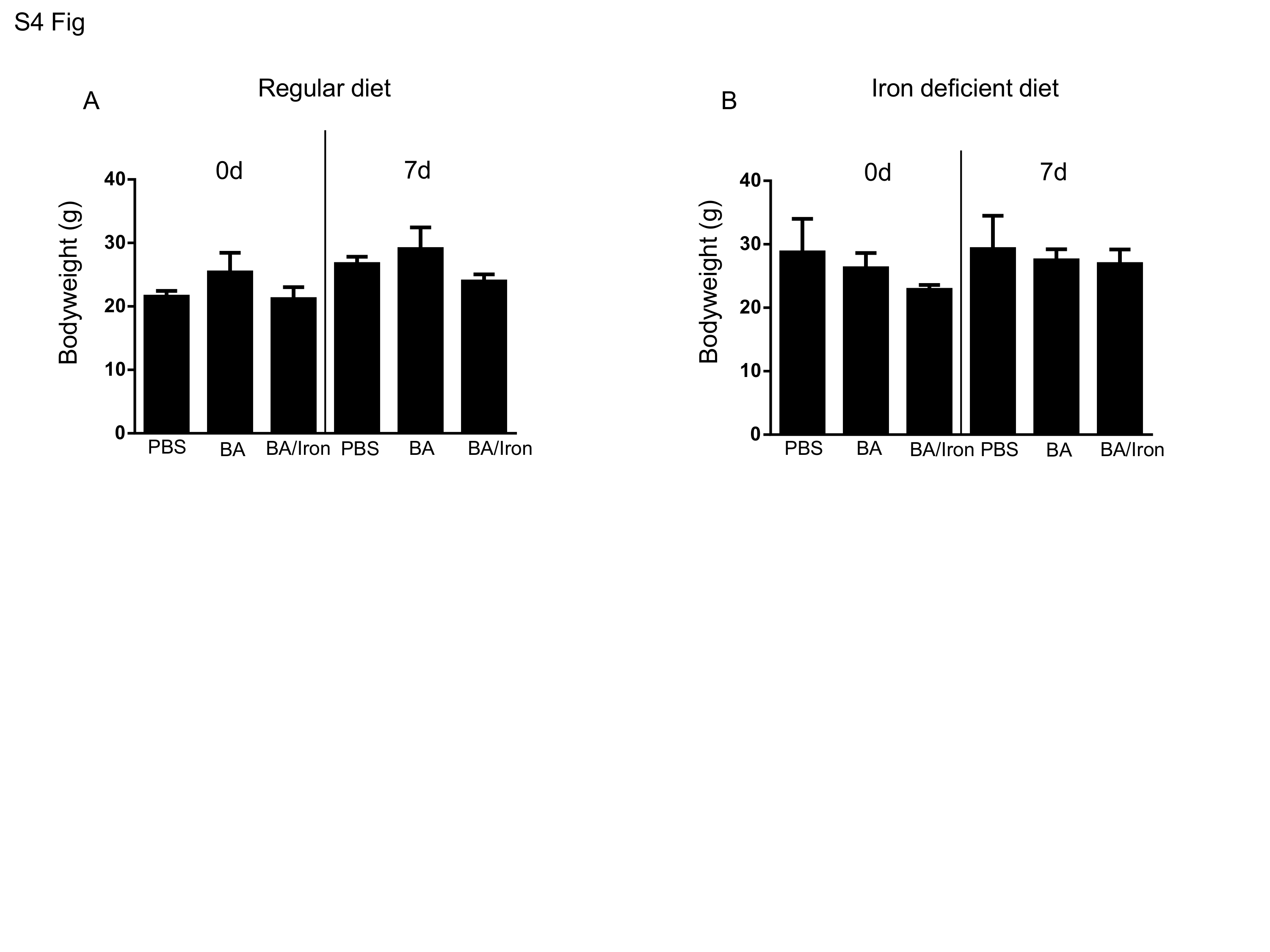

Supplement: S4 Fig — Bodyweight of WT mice were either (A) fed a regular diet (198 ppm iron) or (B) iron deficient diet (5ppm) 4 weeks prior to the administration of 5x10^8^ particles per mouse BA. After 14d mice were treated with a single dose (0.015 mg/g i.v.) iron carboxymaltose. (TIFF) [file pone.0158599.s007.tiff]

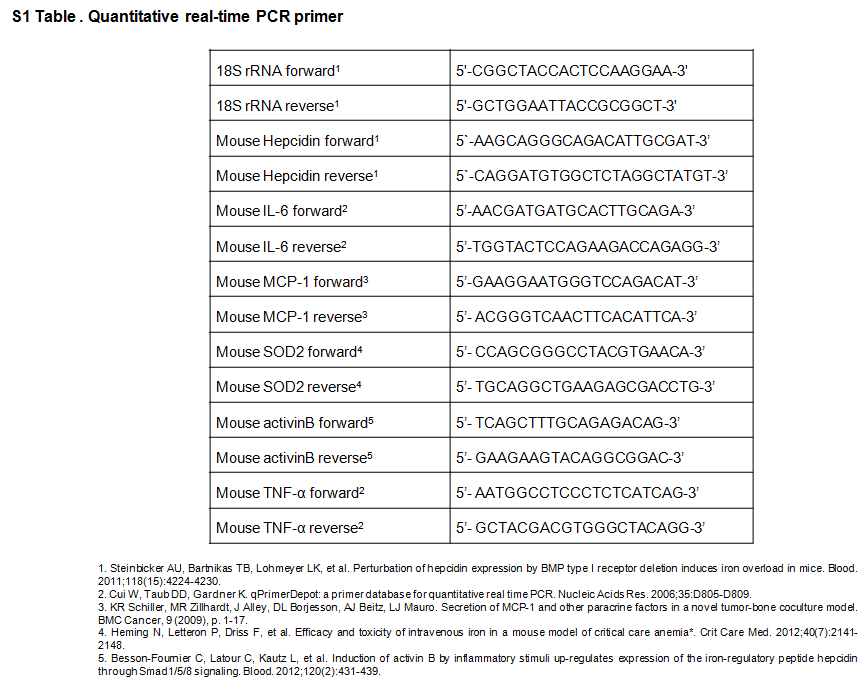

Supplement: S1 Table — (TIF) [file pone.0158599.s008.tif]
